# Is an analysis of copy number variants necessary for various types of kidney ultrasound anomalies in fetuses?

**DOI:** 10.1186/s13039-019-0443-3

**Published:** 2019-07-05

**Authors:** Shaobin Lin, Shanshan Shi, Linhuan Huang, Ting Lei, Danlei Cai, Wenlong Hu, Yi Zhou, Yanmin Luo

**Affiliations:** 1 Fetal Medicine Center, Department of Obstetrics and Gynecology, The First Affiliated Hospital of Sun Yat-sen University, 58 Zhong Shan Er Road, Guangzhou, 510080 Guangdong China; 2 Fetal Medicine Center, The First Affiliated Hospital of Jinan University, Guangzhou, China; 3 Department of Ultrasonic Medicine, The First Affiliated Hospital of Sun Yat-sen University, Guangzhou, China; 4 Clinical Medical Research Center, Shenzhen people’ s hospital, Shenzhen, China

**Keywords:** Copy number variant, Fetus, Kidney anomaly, Ultrasound, Chromosomal microarray analysis

## Abstract

**Background:**

This study aimed to estimate the associations of copy number variants (CNVs) with fetal kidney ultrasound anomalies. A total of 331 fetuses with kidney ultrasound anomalies who underwent prenatal chromosomal microarray analyses were enrolled. The fetuses were classified into groups with isolated and nonisolated anomalies or according to the types of kidney anomalies.

**Results:**

Clinically significant CNVs were identified in 3.4% or 7.3% of fetuses with isolated or nonisolated kidney anomalies, respectively. CNVs were more frequently identified in fetuses with abnormal embryonic migration of the kidneys (6.6%) than in fetuses with malformations of the renal parenchyma (4.7%) or anomalies of the urinary collecting system (3.4%). In particular, CNVs were most frequently detected in fetuses with ectopic kidneys (9.5%) but not in fetuses with horseshoe kidneys or isolated duplex kidneys. Among these CNVs, the most common were del(17)(q12q12) (1.2%) and del(22)(q11q11) (0.6%). The dup(17)(p12p12) and del(15)(q11.2q11.2) CNVs were identified in this study but not in previous studies. The del(X)(p11.4p11.4) and del(16)(p13.3p13.3) CNVs were further implicated as associated with kidney anomalies.

**Conclusions:**

Fetuses with abnormal embryonic migration of the kidneys (particularly ectopic kidneys) showed a higher frequency of clinically significant CNVs, whereas fetuses with horseshoe kidneys or duplex kidneys were less frequently associated with these CNVs.

## Background

Fetal ultrasound scanning during the second or third trimester has permitted the prenatal anatomical assessment of most congenital anomalies of the kidney and urinary tract (CAKUT). Fetal kidney anomalies are common findings in the second trimester ultrasound examination, with a prevalence of ~ 0.1% [1]. These anomalies refer to a broad spectrum of ultrasound diagnoses with diverse severities. Fetuses with the most severe types of kidney anomalies, such as bilateral multicystic dysplastic kidney (MCDK) or renal agenesis, will not survive, while fetuses with other types, such as unilateral mild hydronephrosis or MCDK, may have favorable outcomes. Nevertheless, the prognosis of kidney anomalies also depends on whether they are isolated or nonisolated anomalies and whether they are caused by genetic defects, such as copy number variants (CNVs), which might often lead to additional extrarenal anomalies or neurocognitive impairment [2–4]. In fact, due to the technical limitations and diagnostic accuracy of prenatal imaging, ultrasound diagnoses of fetal kidney anomalies might differ from postnatal ultrasound diagnoses, and thus, prognostic evaluation in prenatal practice might be difficult in some cases, particularly anomalies that are potentially caused by genetic defects [5, 6]. Furthermore, although genetic defect-associated phenotypes might vary considerably (particularly in the prenatal ultrasound findings), which may be attributed to genetic heterogeneity, a genetic diagnosis or exclusion diagnosis would be valuable for further evaluation and management [7–9]. Genetic defects such as pathogenic CNVs and likely pathogenic CNVs have been reported in approximately 4–10% and 3–7% of these cases, respectively [4, 8, 10–13]. These CNVs have mainly been reported to be the causes of congenital malformations of the renal parenchyma, such as renal agenesis and renal dysplasia [11–16]. However, most associated CNVs were identified in patients who were postnatally diagnosed with some type of kidney anomaly, and the contributions of CNVs to the other types of kidney anomalies that are diagnosed prenatally remain unclear; therefore, CNVs associated with various types of fetal kidney ultrasound anomalies would be worth exploring, particularly in prenatal practice.

Although two studies from Shaffer et al. [17] and Donnelly et al. [18] have reported frequencies of pathogenic CNVs or other-than-common benign CNVs (including variants of uncertain significance (VOUS) and pathogenic CNVs) in 6.1% (7/115) or 15.0% (3/20) of fetuses with ultrasound anomalies of the kidney and urinary tract, respectively (the cohort in the study by Shaffer et al. [17] also included fetuses with genital anomalies), the cases in the two studies included only a few subcategories of fetal kidney ultrasound anomalies with a relatively limited number of cases each, and the specific genomic disorders associated with fetal kidney ultrasound anomalies were not described in detail. Overall, clinically significant CNVs associated with fetal kidney ultrasound anomalies have not yet been systematically and exclusively investigated in large cohorts of fetuses with kidney ultrasound anomalies diagnosed prenatally.

Therefore, we performed a retrospective, systematic evaluation of chromosomal abnormalities (≥10 Mb), clinically significant CNVs (< 10 Mb) and absence of heterozygosity (AOH) identified in a cohort of 331 fetuses with kidney ultrasound anomalies using whole-genome chromosomal microarray analysis (CMA). The frequencies of these detected abnormalities were also determined and compared among fetuses with different types of kidney ultrasound anomalies.

## Methods

### Study participants

A consecutive cohort of 331 fetuses with ultrasound anomalies of the kidney who underwent invasive prenatal CMA (most fetuses also underwent karyotyping) from March 2013 to January 2018 at our hospital were included in this retrospective study. Of the 331 pregnant women, 124 women were primiparous, and 207 women were multiparous. The mean maternal age was 29.7 ± 4.5 years (range: 19–44 years). Invasive procedures were performed at a mean of 26.1 ± 3.6 weeks (range: 13–36 weeks). Fetal specimens, including amniotic fluid, chorionic villi and fetal blood, were obtained by invasive chorionic villus sampling (*n* = 1), amniocentesis (*n* = 68), or cordocentesis (*n* = 260), according to the gestational age, and fetal skin tissues were obtained by skin biopsy (*n* = 2) after the termination of pregnancy at 14 and 17 weeks. The mean gestational age for the initial ultrasound evaluation was 24.4 ± 3.6 weeks (range: 13–35 weeks). Among the 331 pregnancies, 302 were conceived naturally, and 29 were conceived via assisted reproductive technology. All patients provided informed consent for the invasive prenatal examinations.

Documented prenatal ultrasound diagnoses of primary kidney anomalies present in the 331 fetuses were reviewed, and these cases were categorized into four groups as described in a previous study: malformations of the renal parenchyma (including renal agenesis, renal dysplasia, MCDK, cystic kidney or polycystic kidney), anomalies of the urinary collecting system (including hydronephrosis or duplex kidney), abnormal embryonic migration of the kidneys (including ectopic kidney or horseshoe kidney) and a combination of kidney anomalies (presence of at least two types of primary kidney anomalies) [19]. The number of cases included in each category described above is summarized in Table 1. The most common ultrasound anomalies in our cohort were malformations of the renal parenchyma (45.0%, 149/331) consisting of renal agenesis and renal dysplasia (*n* = 64), MCDK (*n* = 59), cystic kidney (*n* = 9) and polycystic kidney (*n* = 17). Because the associations between malformations of the renal parenchyma and CNVs have been well described in previous studies, this study did not subclassify this type of kidney anomaly for further analysis [11, 13]. The other less common kidney anomalies were anomalies of the urinary collecting system (35.0%, 116/331), abnormal embryonic migration of the kidneys (18.4%, 61/331) and a combination of kidney anomalies (1.5%, 5/331). Of the 331 fetuses, 62.8% (208/331) had isolated kidney ultrasound anomalies, and 37.2% (123/331) showed nonisolated kidney ultrasound anomalies. The latter group was subclassified into fetuses with kidney ultrasound anomalies plus ultrasound soft markers (*n* = 41) and fetuses with kidney ultrasound anomalies plus additional anomalies (including extrarenal structural anomalies, fetal growth restriction, hydrops fetalis and abnormal amniotic fluid volume; *n* = 82). Furthermore, fetuses with a previously known family history of autosomal recessive or dominant polycystic kidney disease were not included in the study. No fetuses were screened for mutations in any known CAKUT-causing genes before CMA testing.

**Table 1:** Distribution of kidney anomalies in 331 fetuses

Types of fetal kidney anomalies	n	Isolated anomalies n (%)	Nonisolated anomalies n (%)	
			with soft markers	with additional anomalies ^c^
Malformations of the renal parenchyma ^a^	149	93 (62.4)	18 (12.1)	38 (25.5)
Anomalies of the urinary collecting system	116	74 (63.8)	13 (11.2)	29 (25.0)
Hydronephrosis	80	53 (66.3)	6 (7.5)	21 (26.3)
Duplex kidney	36	21 (58.3)	7 (19.4)	8 (22.2)
Abnormal embryonic migration of the kidneys	61	38 (62.3)	10 (16.4)	13 (21.3)
Ectopic kidney	42	25 (59.5)	7 (16.7)	10 (23.8)
Horseshoe kidney	19	13 (68.4)	3 (15.8)	3 (15.8)
Combination of kidney anomalies^b^	5	3 (60.0)	0	2 (40.0)
Total	331	208 (62.8)	41 (12.4)	82 (24.8)

^a^Including renal agenesis, renal dysplasia, multicystic dysplastic kidney, cystic kidney or polycystic kidney

^b^Presence of at least two types of the kidney anomalies described above

^c^Including fetuses with extrarenal structural anomalies, fetal growth restriction, hydrops fetalis or abnormal amniotic fluid volume

### CMA

The CMA was conducted on a single-nucleotide polymorphism (SNP) array platform using CytoScan HD arrays (Affymetrix, Thermo Fisher Scientific, Inc., Waltham, Massachusetts) according to the manufacturer’s protocols. Raw data were analyzed using Chromosome Analysis Suite software (Affymetrix, Thermo Fisher Scientific, Inc., Waltham, Massachusetts), and a threshold of minimal resolution at 100 kb indicated by ≥50 contiguous probes was established to call CNVs. Additionally, AOHs with sizes ≥5 Mb were displayed. The clinical significance of the detected CNVs was determined by reviewing the scientific literature and comparing them with in-house databases and public CNV databases, including the Database of Genomic Variants (http://dgv.tcag.ca/dgv/app/home), Online Mendelian Inheritance in Man (http://www.ncbi.nlm.nih.gov/omim), Database of Genomic Variation and Phenotype in Humans Using Ensembl Resources (https://decipher.sanger.ac.uk/), ClinGen Dosage Sensitivity Map (https://www.ncbi.nlm.nih.gov/projects/dbvar/clingen/index.shtml), ClinVar (https://www.ncbi.nlm.nih.gov/clinvar/) and GeneReviews (https://www.ncbi.nlm.nih.gov/books/NBK1116/). CNVs were classified into five categories, benign CNVs, likely benign CNVs, VOUS, likely pathogenic CNVs and pathogenic CNVs, according to the American College of Medical Genetics and Genomics standards and guidelines for the interpretation of CNVs [20]. Only pathogenic or likely pathogenic CNVs and VOUS were reported in this study. Clinically significant CNVs (< 10 Mb) consisted of pathogenic and likely pathogenic CNVs, and total known pathogenic and likely pathogenic findings consisted of chromosomal abnormalities (≥10 Mb) and clinically significant CNVs in this study. Genes within the CNV or AOH region were defined using the UCSC Genome Browser based on the February 2009 hg19 assembly (http://genome.ucsc.edu/).

### Identification of kidney anomaly-associated CNVs and genes

The associations between the clinically significant CNVs and fetal kidney anomalies were further determined by prioritizing candidate genes for kidney anomaly. We defined the genes as potential drivers for kidney anomaly with a few criteria, including: 1. genes that were suggested to be attributed to kidney anomaly in previous studies; 2. genes expressed in the developing mouse kidney according to the MGI database (http://www.informatics.jax.org/expression.shtml) and the GUDMAP database (https://www.gudmap.org/); 3. genes implicated in the renal phenotypes in mice according to MGI database; 4. genes expressed in the human kidney tissues according to the ProteomicsDB database (https://www.proteomicsdb.org/); and 5. genes that have interactions with known CAKUT-associated genes in protein–protein interaction networks established by the STRING database (https://string-db.org/) [9, 21].

### G-banded karyotyping

G-banded karyotyping was performed using standard procedures. Chromosomal aberrations were identified at a 400-band level using the International System for Human Cytogenetic Nomenclature.

### Statistical analyses

Either the χ^2^ test or Fisher’s exact test was used to compare categorical variables using SPSS software (version 22.0; SPSS Inc., Chicago, Illinois). A *p* value < 0.05 was recognized as indicating statistical significance.

## Results

### CMA results in fetuses with kidney ultrasound anomalies

Overall, abnormal findings were detected in 14.2% (47/331) of fetuses with kidney ultrasound anomalies, while total known pathogenic and likely pathogenic findings were identified in 7.6% (25/331) of these fetuses. Total known pathogenic and likely pathogenic findings were detected in 4.8% (10/208) or 12.2% (15/123) of fetuses with isolated or nonisolated ultrasound anomalies of the kidney (*p* < 0.05), respectively (Table 2). Chromosomal abnormalities and clinically significant CNVs were identified in 2.7% (9/331) and 4.8% (16/331) of fetuses with kidney ultrasound anomalies, respectively, whereas AOH and VOUS were identified in 2.1% (7/331) and 4.5% (15/331) of these fetuses, respectively (Table 2). Furthermore, clinically significant CNVs were identified in 3.4% (7/208) or 7.3% (9/123) of fetuses with isolated or nonisolated ultrasound anomalies of the kidney, respectively (*p* > 0.05). The frequencies of chromosomal abnormalities were 1.4% (3/208) in fetuses with isolated ultrasound anomalies and 4.9% (6/123) in fetuses with nonisolated ultrasound anomalies (*p* > 0.05) (Table 2).

**Table 2:** Characterization of CMA results identified in isolated and nonisolated fetal kidney anomalies

Types of fetal kidney anomalies	n	Abnormal findings	Chromosomal abnormalities (≥10 Mb)			Clinically significant CNVs (< 10 Mb)			Total known pathogenic or likely pathogenic findings ^b^	VOUS	AOH
		n (%)	Aneuploidy	Segmental deletion/duplication	Total (%)	Pathogenic	Likely pathogenic	Total (%)		n (%)	n (%)
Isolated anomalies	208	21 (10.1)	1	2	3 (1.4)	7	0	7 (3.4)	10 (4.8)	8 (3.8)	3 (1.4)
Nonisolated anomalies	123	26 (21.1)	5	1	6 (4.9)	6	3	9 (7.3)	15 (12.2)	7 (5.7)	4 (3.3)
with soft markers	37	8 (21.6)	2	0	2 (5.4)	0	2	2 (5.4)	4 (10.8)	4 (10.8)	0
with additional anomalies ^a^	86	18 (20.9)	3	1	4 (4.7)	6	1	7 (8.1)	11 (12.8)	3 (3.5)	4 (4.7)
Total (%)	331	47 (14.2)	6 (1.8)	3 (0.9)	9 (2.7)	13 (3.9)	3 (0.9)	16 (4.8)	25 (7.6)	15 (4.5)	7 (2.1)

*CNVs* copy number variants, *VOUS* variants of uncertain significance, *AOH* absence of heterozygosity

^a^Including fetuses with extrarenal structural anomalies, fetal growth restriction, hydrops fetalis or abnormal amniotic fluid volume

^b^Including chromosomal abnormalities and clinically significant CNVs

Among the different types of ultrasound anomalies detected in the fetal kidneys, abnormal embryonic migration of the kidneys (9.8% of all cases; 7.9% of isolated cases) exhibited the highest frequency of total known pathogenic and likely pathogenic findings, followed by anomalies of the urinary collecting system (7.8% of all cases; 4.1% of isolated cases) and malformations of the renal parenchyma (6.0% of all cases; 3.2% of isolated cases) (Tables 3). Specifically, clinically significant CNVs were more frequently identified in fetuses with abnormal embryonic migration of the kidneys (6.6% of all cases; 5.3% of isolated cases) than in fetuses with malformations of the renal parenchyma (4.7% of all cases; 3.2% of isolated cases) or anomalies of the urinary collecting system (3.4% of all cases; 1.4% of isolated cases). Notably, fetuses with ectopic kidneys showed the highest frequency (9.5% of all cases; 8.0% of isolated cases) of clinically significant CNVs among the various types of fetal kidney ultrasound anomalies. However, no clinically significant CNVs were detected in fetuses with horseshoe kidneys, whereas no pathogenic CNVs but a likely pathogenic CNV were detected in fetuses with duplex kidneys. Similarly, no chromosomal abnormalities were identified in fetuses with duplex kidneys or ectopic kidneys (Tables 3).

**Table 3:** Comparison of the distribution of chromosomal abnormalities and clinically significant CNVs among different types of fetal kidney anomalies

Types of fetal kidney anomalies	Total								Isolated anomalies				Nonisolated anomalies ^c^			
	n	Chromosomal abnormalities (≥10 Mb)			Clinically significant CNVs (< 10 Mb)			Total ^d^ n (%)	n	Chromosomal abnormalities (≥10 Mb)	Clinically significant CNVs (< 10 Mb)	Total ^d^ n (%)	n	Chromosomal abnormalities (≥10 Mb)	Clinically significant CNVs (< 10 Mb)	Total ^d^ n (%)
		Aneuploidy	Segmental deletion/duplication	Total (%)	Pathogenic	Likely pathogenic	Total (%)									
Malformations of the renal parenchyma ^a^	149	1	1	2 (1.3)	6	1	7 (4.7)	9 (6.0)	93	0	3 (3.2)	3 (3.2)	56	2 (3.6)	4 (7.1)	6 (10.7)
Anomalies of the urinary collecting system	116	3	2	5 (4.3)	2	2	4 (3.4)	9 (7.8)	74	2 (2.7)	1 (1.4)	3 (4.1)	42	3 (7.1)	3 (7.1)	6 (14.3)
Hydronephrosis	80	3	2	5 (6.3)	2	1	3 (3.8)	8 (10.0)	53	2 (3.8)	1 (1.9)	3 (5.7)	27	3 (11.1)	2 (7.4)	5 (18.5)
Duplex kidney	36	0	0	0	0	1	1 (2.8)	1 (2.8)	21	0	0	0	15	0	1 (6.7)	1 (6.7)
Abnormal embryonic migration of the kidneys	61	2	0	2 (3.3)	4	0	4 (6.6)	6 (9.8)	38	1 (2.6)	2 (5.3)	3 (7.9)	23	1 (4.3)	2 (8.7)	3 (13.0)
Ectopic kidney	42	0	0	0	4	0	4 (9.5)	4 (9.5)	25	0	2 (8.0)	2 (8.0)	17	0	2 (11.8)	2 (11.8)
Horseshoe kidney	19	2	0	2 (10.5)	0	0	0	2 (10.5)	13	1 (7.7)	0	1 (7.7)	6	1 (16.7)	0	1 (16.7)
Combination of kidney anomalies ^b^	5	0	0	0	1	0	1 (20.0)	1 (20.0)	3	0	1 (33.3)	1 (33.3)	2	0	0	0
Total (%)	331	6	3	9 (2.7)	13	3	16 (4.8)	25 (7.6%)	208	3 (1.4)	7 (3.4)	10 (4.8)	123	6 (4.9)	9 (7.3)	15 (12.2)

*CNVs* copy number variants

^a^Including renal agenesis, renal dysplasia, multicystic dysplastic kidney, cystic kidney or polycystic kidney

^b^Presence of at least two types of the kidney anomalies described above

^c^Including fetuses with soft markers and fetuses with extrarenal structural anomalies, fetal growth restriction, hydrops fetalis or abnormal amniotic fluid volume

^d^Including chromosomal abnormalities and clinically significant CNVs

### Clinically significant CNVs detected in fetuses with kidney ultrasound anomalies

This study identified 16 fetuses carrying clinically significant CNVs, including 13 fetuses carrying pathogenic CNVs and 3 fetuses carrying likely pathogenic CNVs (Table 4). Of the 16 fetuses, 13 fetuses carried 1 clinically significant CNV, and the other 3 fetuses carried 2 clinically significant CNVs. Of these 3 fetuses, 1 fetus carried 1 likely pathogenic CNV and 1 pathogenic CNV (case 12), and 2 fetuses carried pathogenic CNVs (cases 7 and 9). Nineteen clinically significant CNVs were detected in these 16 fetuses, and the sizes of the CNV distributions ranged from 0.11 Mb to 6.89 Mb. Among the 19 CNVs, 14 were copy number losses, and 5 were copy number gains. Parent studies were performed in 7 cases, and the results showed that 6 CNVs were de novo deletions and 1 CNV was a maternally inherited deletion. Among the 16 fetuses, 7 (43.7%) showed isolated ultrasound anomalies of the kidney (including ectopic kidney, hydronephrosis and MCDK), and 9 (56.3%) showed nonisolated ultrasound anomalies of the kidney (7 with additional anomalies and 2 with soft markers). Notably, the CNVs in the 16 fetuses mainly contributed to ectopic kidney, hydronephrosis, and malformations of the renal parenchyma (renal dysplasia or MCDK).

**Table 4:** Characteristics of clinically significant CNVs identified in 16 fetuses with ultrasound anomalies of the kidney

Case	Genomic disorder	CNV ^a^	Type	Size (Mb)	Clinical significance	Kidney anomaly	Urinary tract anomaly	Extrarenal anomaly	Karyotype
1	RCAD syndrome	17q12(34477479_36397323) × 1	del	1.92	Pathogenic	MCDK (R)	–	–	46, XX
2	RCAD syndrome	17q12(34822465_36283612) × 1 mat	del	1.46	Pathogenic	Hydronephrosis (L) + MCDK (R)	Dilated ureter (L)	–	46, XY
3	RCAD syndrome	17q12(34477479_36410559) × 1 dn	del	1.93	Pathogenic	Bilateral hydronephrosis	–	–	46, XY
4	RCAD syndrome	17q12(34822465_36404138) × 1 dn	del	1.58	Pathogenic	Bilateral MCDK	–	–	46, XY
5	22q11 deletion syndrome	22q11.21(18916842_21800471) × 1 dn	del	2.88	Pathogenic	Ectopic kidney (L)	Ureterocele	–	46, XX
6	22q11 deletion syndrome	22q11.21(18916842_21798907) × 1 dn	del	2.88	Pathogenic	Bilateral renal dysplasia	–	Truncus arteriosus, right aortic arch, absent ductus arteriosus, short penis, thickened nuchal fold,	46, XY
7	Wolf-Hirschhorn syndrome/8p23.1-pter duplication	4p16.3(68345_3950060) × 1, 8p23.3p23.1(158048_7044046) × 3	del/dup	3.88/6.89	Pathogenic/ pathogenic	Bilateral renal dysplasia	–	FGR, persistent left superior vena cava, absent nasal bone	46, XX, der(4)t(4;8) (p16.3;p23.2)
8	Rubinstein-Taybi syndrome	16p13.3(3827552_3935836) × 1	del	0.11	Pathogenic	Ectopic kidney (R)	–	–	46, XX
9	20q13.33-qter duplication/Phelan-Mcdermid syndrome	20q13.33(60862389_62915555) × 3, 22q13.31q13.33(44465713_51197838) × 1	dup/del	2.05/6.70	Pathogenic/ pathogenic	MCDK (L)	–	–	46, XY
10	Sotos syndrome	5q35.2q35.3(175570677_177469711) × 1 dn	del	1.90	Pathogenic	Ectopic kidney (R)	–	FGR, bilateral mild ventriculomegaly	46, XX
11	Williams-Beuren syndrome	7q11.23(72701018_74141746) × 1	del	1.44	Pathogenic	Ectopic kidney (R)	–	FGR	46, XX
12	Bardet-Biedl syndrome 3/ Charcot-Marie-Tooth syndrome type 1A (CMT1A)	3q11.2(97414335_97520233) × 3, 17p12(14108911_15473312) × 3	dup/dup	0.11/1.36	Likely pathogenic /pathogenic	Bilateral renal dysplasia	–	FGR, oligohydramnios, absent end-diastolic velocity in umbilical artery	Unknown
13	Proximal 6q deletion	6q14.2q15(84775390_89494504) × 1 dn	del	4.72	Pathogenic	Hydronephrosis (R)	–	Persistent left superior vena cava, mild ventriculomegaly (R), cavum velum interpositum cyst	46, XX
14	16p13.11 recurrent microduplication (neurocognitive disorder susceptibility locus)	16p13.11(15936927_16146987) × 3	dup	0.21	Likely pathogenic	Hydronephrosis (R)	–	Echogenic intracardiac foci	46, XY
15	15q11.2 recurrent deletion (BP1-BP2)	15q11.2(22770421_23282799) × 1	del	0.51	Likely pathogenic	Duplex kidney (L)	Dilated ureter (L)	Echogenic intracardiac foci	46, XX, 22pss
16	Mental retardation and microcephaly with pontine and cerebellar hypoplasia	Xp11.4(41749904_41998547) × 1	Loss	0.25	Likely pathogenic	Bilateral renal dysplasia	–	FGR, micrognathia, persistent left superior vena cava, posterior fossa cyst, cerebellar hypoplasia, Blake’s pouch cysts, thickened subcutaneous soft tissue	46, XX

*CNV* copy number variant, *MCDK* multicystic dysplastic kidney, *L* left, *R* right, *FGR* fetal growth restriction, *mat* maternal, *dn* de novo, *del* deletion, *dup* duplication

^a^CNV positions are based on the UCSC genome build hg19

### Identification of kidney anomaly-associated CNVs and genes

With the use of a gene prioritization approach based on public databases and bioinformatic tools, this study identified 6 novel and 5 known genes associated with fetal kidney anomaly from 10 clinically significant CNVs (Table 5). However, candidate genes could not be identified among the other 5 clinically significant CNVs, including deletions of 4p16.3, 5q35.2q35.3 and 7q11.23 and duplications of 8p23.1p23.3 (encompassing ~ 14 OMIM genes) and 20q13.33 (encompassing ~ 44 OMIM genes). Of note, the deletions of 4p16.3, 5q35.2q35.3 and 7q11.23 contributing to Wolf-Hirschhorn syndrome, Sotos syndrome and Williams-Beuren syndrome, respectively, were also considered to be associated with kidney anomaly.

**Table 5:** Identification of genes associated with kidney anomaly

Gene	Corresponding CNV		Reported kidney anomalies in previous studies	Expressed in the developing mouse kidney	Renal phenotype in mouse	Expressed in the human kidney or cell lines	Involved in protein–protein interaction network with known CAKUT-associated genes	Association with kidney anomaly
	Chromosomal region	Type						
*HNF1B*	17q12	del	+	+	+	+	+	Known
*TBX1, CRKL*	22q11.21	del	+	+	–	+	+	Known
*CREBBP*	16p13.3	del	+	+	–	+	+	Novel
*SHANK3*	22q13.31q13.33	del	+	+	–	+	–	Novel
*ARL6*	3q11.2	dup	+	–	–	+	+	Known
*PMP22*	17p12	dup	–	–	–	+	–	Novel
*TBX18*	6q14.2q15	del	+	+	+	+	+	Known
*MYH11*	16p13.11	dup	+	+	+	+	+	Novel
*NIPA1*	15q11.2	del	–	+	–	+	–	Novel
*CASK*	Xp11.4	del	+	+	–	+	–	Novel

*CNV* copy number variant, *CAKUT* congenital anomalies of the kidney and urinary tract, *del* deletion, *dup* duplication

Among these clinically significant CNVs, the most frequent CNV was a 17q12 recurrent deletion associated with renal cyst and diabetes (RCAD) syndrome (1.2%, 4/331), accounting for 21.1% (4/19) of all detected clinically significant CNVs or 26.7% (4/15) of all detected pathogenic CNVs. The second most frequent CNV was a 22q11 deletion associated with 22q11 deletion syndrome (0.6%, 2/331), contributing to 10.5% (2/19) of detected clinically significant CNVs or 13.3% (2/15) of all detected pathogenic CNVs. The other clinically significant CNVs were identified only once in this study (Table 4). Of these CNVs, both a 16p13.3 deletion and an Xp11.4 deletion were single-gene deletions, encompassing the *CREBBP* and *CASK* genes, respectively. The Xp11.4 deletion resulted in loss of the first exon and 5′ UTR region of the *CASK* gene, which is associated with a known X-linked dominant genetic syndrome of mental retardation and microcephaly with pontine and cerebellar hypoplasia. A 15q11.2 deletion associated with neurodevelopmental disease and a 17p12 duplication associated with Charcot-Marie-Tooth syndrome type 1A (CMT1A) were also identified in this study. To the best of our knowledge, neither the 15q11.2 deletion nor the 17p12 duplication have been previously reported to be associated with kidney anomalies.

### Comparison of abnormal results from CMA and G-banded karyotyping

Of the 331 fetuses, 323 fetuses also underwent karyotyping, with a success rate of 99.4% (321/323) and failure in 2 cases. Then, we compared the detection efficiencies between CMA and karyotyping in 321 fetuses (not including 1 fetus carrying 1 AOH that failed karyotyping and another 2 fetuses that did not receive karyotyping in which 1 pathogenic CNV and 1 VOUS were present, respectively). Overall, CMA revealed increasing yields of clinically significant CNVs, VOUS and AOH in 4.4% (14/321), 4.4% (14/321) and 1.9% (6/321) of fetuses with ultrasound anomalies of the kidney, respectively, compared with karyotyping (Table 6).

**Table 6:** Comparison of abnormal results from CMA and karyotyping in 321 fetuses

Abnormal results	n	Detected with karyotyping	Detected with CMA
		n (%)	n (%)
Chromosomal abnormalities (≥10 Mb) ^a^	10	10 (100)	9 (90.0)
Aneuploidy	6	6 (100)	6 (100)
45, X	2	2	2
47, XXX	1	1	1
47, XY, + 21	1	1	1
47, XY, + 13	1	1	1
45, X[18]/46, X, Yqh-[19]	1	1	1
Segmental deletion/duplication	3	3 (100)	3 (100)
46, XY, der(22)t(Y;22)(q11;q13.3)	1	1	1
46, XY, der(9)t(2;9)(q37;q34)	1	1	1
47, XY, +mar[8]/46,XY[30] ^b^	1	1	1
Balanced inversion	1	1 (100)	0
46, XY, inv.(20) (p13q13.1) mat	1	1	0
Clinically significant CNVs (< 10 Mb) ^c^	15	1 (6.7)	15 (100)
VOUS (< 10 Mb) ^c^	14	0	14 (100)
AOH (≥10 Mb) ^d^	6	0	6 (100)
Total	45	11 (24.4)	44 (97.8)

*CMA* chromosomal microarray analysis, *CNVs* copy number variants, *VOUS* variants of uncertain significance, *AOH* absence of heterozygosity

^a^Described based on karyotype results

^b^CMA showed a mosaic duplication with a size of 20.98 Mb: arr[hg19] 2p11.2q12.1(83611838_1045 94,881) × 2.46

^c^2 fetuses that did not undergo karyotyping were excluded

^d^1 fetus that failed karyotyping was excluded

## Discussion

CMA has been recommended as the first-tier test over karyotyping for fetuses with structural anomalies, and it has identified ~ 6.0% of clinically significant CNVs in fetuses with normal karyotypes [22]. However, CNVs associated with fetal kidney ultrasound anomalies have not been systematically and exclusively investigated in prenatal practice. Thus, this study performed a retrospective analysis of the associations between CNVs and chromosomal abnormalities detected by CMA and kidney ultrasound anomalies present in a cohort of 331 fetuses.

Consequently, CMA revealed an increasing yield of clinically significant CNVs in 4.4% (14/321) of fetuses with kidney ultrasound anomalies compared with karyotyping. Thus, CMA was more valuable than karyotyping for these fetuses. Furthermore, clinically significant CNVs were identified in 3.4% (7/208) of fetuses with isolated ultrasound anomalies of the kidney, a lower value than previously reported frequencies in other specific anatomical systems, such as the musculoskeletal (~ 8%), cardiac (~ 7%) or central nervous systems (~ 6%) [17, 23, 24]. A systematic review by de Wit MC et al. [23] indicated that pathogenic CNVs were present in 5.9% (95% CI, 2.2–9.6%) of fetuses with isolated ultrasound anomalies of the urogenital system, but the included studies analyzed fetuses with ultrasound anomalies of not only the urinary system but also the genital system, and both the types of ultrasound anomalies and the number of included cases were limited. The current study might define a more representative frequency of clinically significant CNVs in fetuses with isolated ultrasound anomalies of the kidney.

In the groups of ultrasound anomalies of the kidney, clinically significant CNVs were more frequently identified in fetuses with abnormal embryonic migration of the kidneys than in fetuses with either malformations of the renal parenchyma or anomalies of the urinary collecting system. Interestingly, the frequencies of clinically significant CNVs in cases with malformations of the renal parenchyma reported in the current study (4.7%, 7/149) and the studies from Caruana et al. [13] (2.3%, 1/43) and Sanna-Cherchi et al. (10.5%, 55/522) disagreed [11]. This discrepancy might be attributed to the differences in sample sizes and case selection bias in these studies, as well as the differences in the diagnoses of kidney anomalies between prenatal and postnatal cases. Nevertheless, the present study may suggest a prenatal yield of clinically significant CNVs leading to malformations of the renal parenchyma present in fetuses. Additionally, ectopic kidney showed the highest frequency (9.5%) of clinically significant CNVs among various types of fetal kidney ultrasound anomalies. Regarding fetuses with isolated ultrasound anomalies of the kidney, clinically significant CNVs were revealed only in fetuses with ectopic kidneys (8.0%), malformations of the renal parenchyma (3.2%) and hydronephrosis (1.9%).

In our cohort, 19 clinically significant CNVs were identified in 16 fetuses with kidney anomalies, including ectopic kidney, hydronephrosis and malformations of the renal parenchyma. These CNVs are associated with known genomic disorders or neurodevelopmental disorders. Among these CNVs, both a 17q12 deletion and a 22q11 deletion were common pathogenic CNVs. The high frequencies of the 17q12 deletion (1.2%) and 22q11 deletion (0.6%) observed in our cohort were consistent with the results reported by other postnatal studies, suggesting that they were the most common genomic disorders in either prenatal or postnatal individuals with kidney anomalies, with frequencies of 2.2 and 1.1% in postnatal cases, respectively [11, 25]. The 16p13.11 recurrent region (including *MYH11*) was identified as a neurocognitive disorder susceptibility locus. Either deletions or duplications in this region were reportedly associated with kidney anomalies in previous studies [11, 13]. The dup(16)(p13.11p13.11) encompassing the partial *MYH11* gene in case 14 overlapped only with the partial 16p13.11 recurrent region (including *MYH11*); therefore, a likely pathogenic classification was defined in this study. In addition, both del(16)(p13.3p13.3) and del(X)(p11.4p11.4) were single-gene deletions, encompassing part of the *CREBBP* and *CASK* genes, respectively. *CREBBP* encodes a histone acetyltransferase that activates transcription by controlling the interaction between DNA-binding transcription factors and the RNA polymerase II complex, contributing to embryonic development and cell processes (such as growth differentiation, DNA repair and apoptosis) [26, 27]. Partial or complete deletion of the *CREBBP* gene was reported to be responsible for Rubinstein-Taybi syndrome, which is characterized by intellectual disability, postnatal growth deficiency, microcephaly and distinctive facial features [28]. However, kidney anomalies, including previously reported renal agenesis, renal hypoplasia and renal pelvis ectasia, were less common phenotypes [29–32]. The *CASK* gene was recognized as sensitive to haploinsufficiency and was mainly associated with developmental phenotypes. Loss of function mutations in the gene are attributed to X-linked dominant intellectual disability and microcephaly with pontine and cerebellar hypoplasia. Kidney anomalies (such as fusion of the kidneys and rotated kidneys) have been observed in a few patients with *CASK* mutations [33]. However, kidney phenotypes in mice were not believed to be caused by *CASK*-null mutations alone but instead by a cooperative effect between *CASK* and other genes, such as *Dlg1* [34]. The correlations between the *CASK* gene and renal phenotypes remain unclear. Nevertheless, we postulate that *CREBBP* and *CASK* are candidate genes associated with kidney anomalies with incomplete penetrance and variable expressivity. In case 12, a dup (3) (q11.2q11.2) and a dup (17) (p12p12) were identified. Moreover, the 17p12 recurrent (HNPP/CMT1A) region (including *PMP22*) has been attributed to hereditary neuropathy with liability to pressure palsies (HNPP) caused by the del (17) (p12p12) and CMT1A caused by the dup(17) (p12p12). Although HNPP was identified in two patients with CAKUT in two previous studies, and CMTIA was observed only in the current study, both findings indicated that the 17p12 recurrent region might be a sensitive region contributing to kidney anomalies [4, 13]. Furthermore, the dup (3) (q11.2q11.2) region encompassed part of the *ARL6* gene. Mutations in this gene may cause Bardet-Biedl syndrome 3, which contributes to renal structural anomalies, such as renal hypoplasia. Although the *ARL6* gene is associated with an autosomal recessive phenotype, we were unable to exclude the potential interaction between dup (3) (q11.2q11.2) and dup (17) (p12p12) as a cause of kidney anomalies. A 15q11.2 recurrent deletion (BP1-BP2) was identified as a susceptibility locus for neurodevelopmental disorders with reduced penetrance. This deletion has been defined as a likely pathogenic CNV and the association between del (15) (q11.2q11.2) and renal phenotypes remains unclear. Del (6) (q14.2q15) is an extremely rare CNV. This CNV has been observed in a few patients with kidney anomalies, and the *TBX18* gene has been suggested as a candidate gene associated with kidney anomalies [35]. Other pathogenic CNV-associated genomic disorders, including Williams-Beuren syndrome, Sotos syndrome and Phelan-Mcdermid syndrome, have often been referred to as multiple congenital anomalies, and related kidney anomalies have also been reported in previous studies [36–38]. This study identified a series of clinically significant CNVs associated with genomic or neurodevelopmental disorders as causes of ultrasound anomalies of the fetal kidney, indicating that embryonic nephrogenesis was susceptible to these genomic imbalances. In summary, duplication at 17p12 and deletions at Xp11.4, 16p13.3 and 15q11.2 were further implicated as associated with fetal kidney anomalies.

Additionally, although this study included the majority of prenatal ultrasound diagnoses of kidney anomalies in the analysis, notably, not all kidney anomalies are efficiently detected by a fetal ultrasound examination, and not all prenatal ultrasound diagnoses of kidney anomalies are consistent with postnatal diagnoses; thus, certain discrepancies between this and other postnatal studies are unavoidable. Therefore, prospective studies with postnatal follow-up for these prenatally diagnosed cases should be performed to determine potential discrepancies.

## Conclusions

In summary, prenatal CMA revealed a higher frequency of clinically significant CNVs than chromosomal abnormalities in fetuses with kidney ultrasound anomalies (either isolated or nonisolated anomalies). Clinically significant CNVs were more frequently identified in fetuses with abnormal embryonic migration of the kidneys than in fetuses with either malformations of the renal parenchyma or anomalies of the urinary collecting system. In particular, fetuses with ectopic kidneys showed the highest frequency of clinically significant CNVs, whereas fetuses with horseshoe kidneys or duplex kidneys were less frequently associated with clinically significant CNVs.

## Data Availability

The datasets used and/or analyzed during the current study are available from the corresponding author on reasonable request.

## Abbreviations

AOH: Absence of heterozygosity
CAKUT: Congenital anomalies of the kidney and urinary tract
CMA: Chromosomal microarray analysis
CNVs: Copy number variants
MCDK: Multicystic dysplastic kidney
RCAD: Renal cyst and diabetes
VOUS: Variants of uncertain significance
